# Hydrogel Delivery Device for the In Vitro and In Vivo Sustained Release of Active rhGALNS Enzyme

**DOI:** 10.3390/ph16070931

**Published:** 2023-06-27

**Authors:** Michael Flanagan, Qi Gan, Saahil Sheth, Rachel Schafer, Samuel Ruesing, Linda E. Winter, Karoly Toth, Silviya P. Zustiak, Adriana M. Montaño

**Affiliations:** 1 Department of Pediatrics, School of Medicine, Saint Louis University, St. Louis, MO 63104, USA; 2 Department of Biomedical Engineering, Saint Louis University, St. Louis, MO 63103, USA; 3 School of Medicine, Saint Louis University, St. Louis, MO 63104, USA; 4 Department of Microbiology and Molecular Immunology, School of Medicine, Saint Louis University, St. Louis, MO 63104, USA; 5 Department of Biochemistry and Molecular Biology, School of Medicine, Saint Louis University, St. Louis, MO 63104, USA

**Keywords:** polyethylene glycol hydrogel, drug delivery, sustained release, Morquio A, GALNS

## Abstract

Morquio A disease is a genetic disorder resulting in N-acetylgalactosamine-6-sulfate sulfatase (GALNS) deficiency, and patients are currently treated with enzyme replacement therapy via weekly intravenous enzyme infusions. A means of sustained enzyme delivery could improve patient quality of life by reducing the administration time, frequency of hospital visits, and treatment cost. In this study, we investigated poly(ethylene-glycol) (PEG) hydrogels as a tunable, hydrolytically degradable drug delivery system for the encapsulation and sustained release of recombinant human GALNS (rhGALNS). We evaluated hydrogel formulations that optimized hydrogel gelation and degradation time while retaining rhGALNS activity and sustaining rhGALNS release. We observed the release of active rhGALNS for up to 28 days in vitro from the optimized formulation. rhGALNS activity was preserved in the hydrogel relative to buffer over the release window, and encapsulation was found to have no impact on the rhGALNS structure when measured by intrinsic fluorescence, circular dichroism, and sodium dodecyl sulfate-polyacrylamide gel electrophoresis (SDS-PAGE). In vivo, we monitored the retention of fluorescently labeled rhGALNS in C57BL/6 albino mice when administered via subcutaneous injection and observed rhGALNS present for up to 20 days when delivered in a hydrogel versus 7 days in the buffer control. These results indicate that PEG hydrogels are suitable for the encapsulation, preservation, and sustained release of recombinant enzymes and may present an alternative method of delivering enzyme replacement therapies that improve patient quality of life.

## 1. Introduction

Mucopolysaccharidosis IVA (MPS IVA, Morquio A) is an autosomal recessive disorder characterized by a functional deficiency in the N-acetylgalactosamine-6-sulfate sulfatase (GALNS) enzyme [1]. It occurs in approximately one in every 200,000 to 250,000 live births [2,3]. The GALNS enzyme is responsible for breaking down specific glycosaminoglycan (GAG) substrates in the body, and its deficiency results in a systemic buildup of GAGs, specifically in chondroitin-6-sulfate (C6S) and keratan sulfate (KS) [4]. The symptoms of patients with Morquio A include short stature, skeletal deformities, laxity of joints, corneal clouding, and heart valvular defects [5,6].

The treatment of Morquio A disease primarily focuses on the reduction in GAGs buildup and the management of symptoms. The gold-standard treatment for this patient population is a weekly intravenous (i.v.) infusion of the recombinant human GALNS enzyme (rhGALNS), also known as enzyme replacement therapy (ERT) [7,8]. This treatment has been effective in lowering GAG levels in blood and urine as well as improving other endpoints, such as the 6 min walk test [7,9]. ERT comes with several limitations, including a short duration of efficacy. Currently, ERT is conducted via time-intensive weekly i.v. infusions, which consist of large amounts of the rhGALNS enzyme administered over 4–5 h. Much of the infused enzyme is quickly taken up by the liver and metabolized [4,10]. As a result, a significant amount of the treatment is lost before any benefit is provided. In addition, unlike enzymes associated with other MPS disorders, GALNS has a short half-life [4,10]. Lastly, treatment with functional rhGALNS induces an immune response in Morquio A patients, resulting in neutralizing antibodies that can interfere with the outcome of the treatment, as commonly seen in ERTs [7,11]. Thus, the administration of immunosuppressants is required.

There is an urgent need to develop novel drug delivery methods that can address the limitations of the current treatment for Morquio A patients. Improvement areas include the distribution of rhGALNS at a low and sustained dose and new protocols that can decrease the immune response to rhGALNS. Using hydrogel delivery devices, such as poly-ethylene glycol (PEG) hydrogels, presents an interesting approach. PEG polymers are already widely used for in vivo treatments and are FDA-approved for use in medical devices [12,13]. In this study, we prepared hydrogels using a Michael-type addition between a PEG-dithiol and a multi-arm PEG-acrylate or a vinyl sulfone. We chose this chemistry because it is highly specific and allows for protein encapsulation prior to gelation [14]. The time-gelation mechanism also allows for the preparation of injectable hydrogel formulations [15,16]. Others have tested similar Michael-type addition chemistry between thiol- and acrylate-containing PEG polymers. For example, an injectable formulation used to deliver RANTES in vivo released active chemokine for up to 2 weeks [17]. Additionally, β-galactosidase has been delivered in in vitro models of gangliosides via injectable PEG-polylactic acid polymersomes [18]. However, such delivery models have not been investigated for MPS diseases.

Our previous study demonstrated that active rhGALNS can be encapsulated within PEG hydrogels and can be slowly released over one week and uptaken by deficient cells in vitro [19]. Here, we studied the application of these hydrogel formulations in vivo for their compatibility with rhGALNS, their gelation time, and their in vivo degradability. Enzyme release profiles for the various hydrogel formulations were tested in vitro for up to 36 days and in vivo for up to 24 days to show the retention of enzyme activity.

## 2. Results

### 2.1. PEG Hydrogel Gelation and Degradation In Vitro Are Dependent on Reaction pH and Polymer Concentration

**Figure 1:**
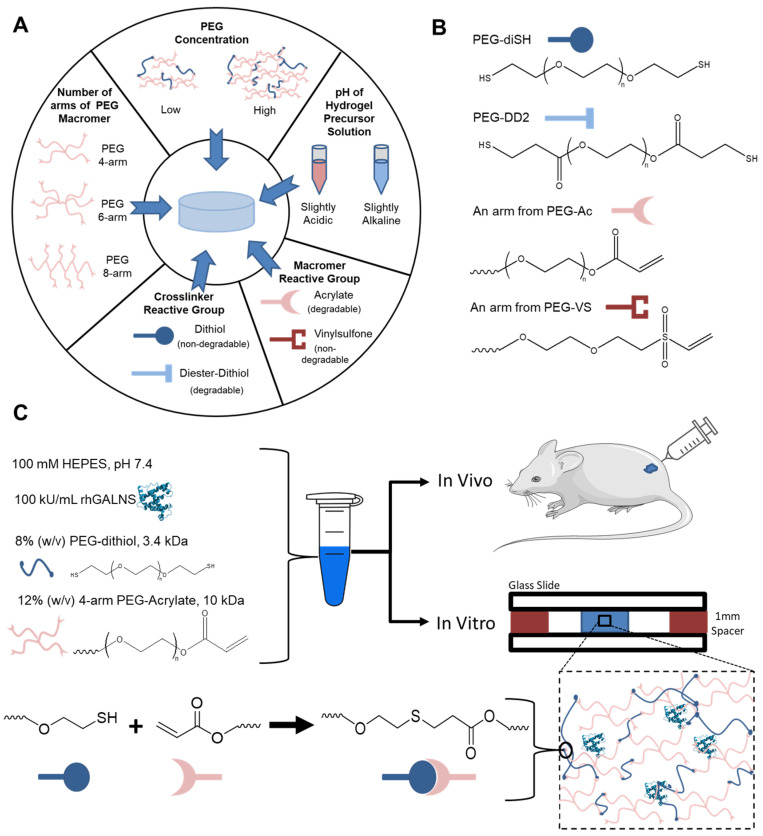
(**A**) Schematic representation of the conditions tested for their effect on hydrogel properties. (**B**) Chemical structures for a polyethylene glycol-dithiol (PEG-diSH) crosslinker, a polyethylene glycol-diester-dithiol (PEG-DD2) crosslinker, an arm of the four-, six-, or eight-arm polyethylene glycol-acrylate macromer, and an arm of the four-, six-, or eight-arm polyethylene glycol-vinylsulfone macromer. (**C**) Example Michael-type addition gelation reaction for a 20% *w*/*v* PEG hydrogel formed from the four-arm PEG Acrylate, 10 kDa, and PEG-diSH, 3.4 kDa.

**Figure 2:**
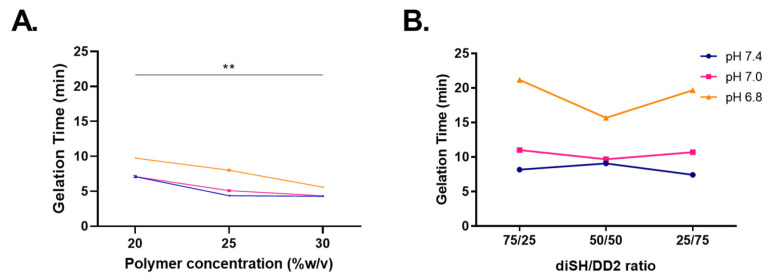
(**A**) Hydrogel gelation time as a function of PEG polymer concentration. Hydrogels were prepared with an eight-arm PEG-Ac macromer and PEG-diSH crosslinker. (**B**) Hydrogel gelation time as a function of the crosslinker PEG-diSH:PEG-DD2 ratio. Hydrogels were prepared with four-arm PEG-VS and were 20% *w*/*v* in the total polymer concentration. The pH values refer to the pH of the hydrogel precursor solution. Data are the mean ± SD, *n* = 3. ** *p* ≤ 0.01.

Here, we tested several hydrogel formulations for their effect on hydrogel gelation and degradation in vitro (Figure 1). Gelation time has implications for hydrogel in vivo injectability and even degradability. We have previously shown that, for the PEG hydrogels tested here, gelation time affects degradability, with fast gelation leading to fast degradation and vice versa [20]. We first varied the pH of the hydrogel precursor solution (pH of 6.8, 7.0, and 7.4) and PEG polymer concentration (20%, 25%, and 30% *w*/*v*) for hydrogels made of an eight-arm PEG-Ac macromer and PEG-diSH crosslinker [20] (Figure 2A). Overall, the gelation time varied between 5 and 10 min and decreased with an increase in the polymer concentration or solution pH. Gelation was slower at a pH of 6.8, but there was no significant difference in gelation time between pH 7.0 and 7.4 (*p* = 0.051). A higher polymer concentration led to faster gelation for any pH tested. However, at the highest pH of 7.4, no further decrease in the gelation time was noted between 25% and 30% *w*/*v* polymer concentrations. Overall, there was a significant difference between different polymer concentrations (*p* = 0.007).

We next tested hydrogels prepared with the four-arm PEG-Ac and varying ratios of the crosslinkers PEG-diSH (diSH, non-degradable) and PEG-diester-dithiol (DD2, degradable) (Figure 2B). As before, the gelation time decreased with the increase in the reaction pH. We did not expect major differences with respect to the crosslinker ratio, as both crosslinkers independently have similar gelation times (*p* = 0.46) [20]. However, the crosslinker ratio of 1:1 showed the least dependence on pH, with a gelation time lower than other ratios at the low pH of 6.8. Overall, the gelation time for hydrogels prepared with the four-arm PEG-Ac varied between 7 and 22 min, which was higher than the gelation time for the eight-arm PEG-Ac of 5–10 min at the same pH (Figure 2A). 

In summary, we noted that the gelation time decreased with an increase in the PEG macromer number of arms, with an increase in the polymer concentration, and with a decrease in the hydrogel precursor solution pH (*p* = 0.02). A subset of these experiments was performed by preparing the precursor solution in a syringe and injecting it into the microcentrifuge tube to mimic the shear stress of a subcutaneous injection (Figure S1). Similar trends were observed between the two experiments.

### 2.2. Hydrogel Degradation In Vivo following Subcutaneous Injection

**Figure 3:**
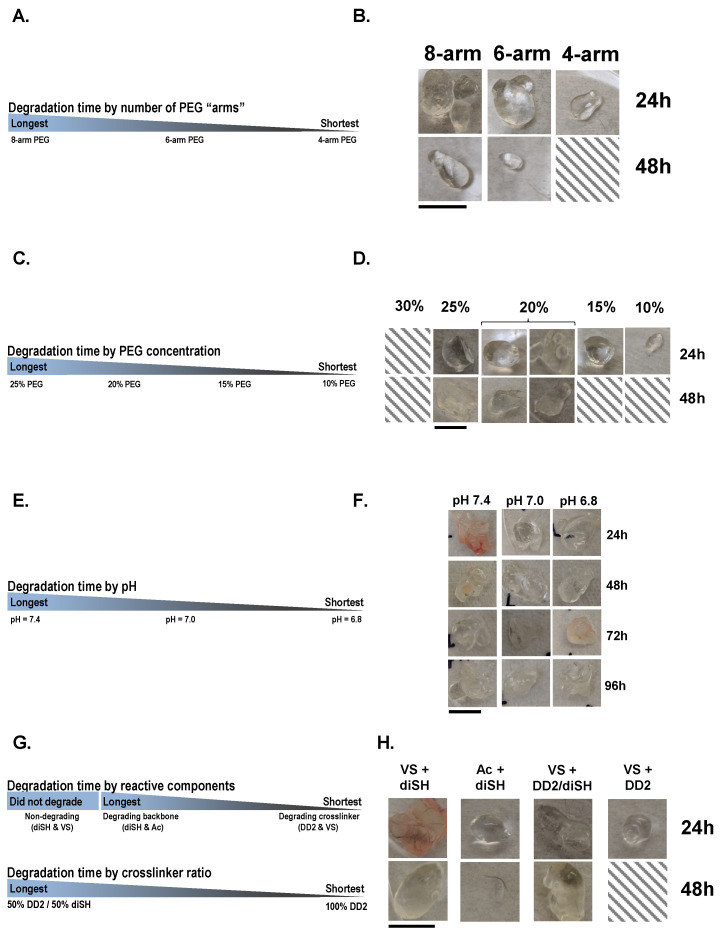
Factors affecting hydrogel degradation in vivo. Changes in the degradation profile were assessed upon manipulating the: (**A**,**B**) number of PEG arms, (**C**,**D**) PEG concentration, (**E**,**F**) reaction pH, and (**G**,**H**) hydrogel composition. Scale bar = 10 mm.

Following in vivo injections, we removed the hydrogels every 24 h, up to 96 h (Figure 3). We first made 10% *w*/*v* PEG gels using a PEG-diSH crosslinker, varying the number of arms of the multi-arm PEG-Ac macromer. We observed that, correlating with a faster gelation time, increasing the number of arms resulted in gels that were slower to degrade in vivo (Figure 3A,B). However, at a lower PEG concentration (10–15% *w*/*v*), all gels had completely degraded after 48 h. Complementing the effect of the number of arms in the macromer, we showed that increasing the polymer concentration increased the longevity of the gels in vivo (Figure 3C,D). All hydrogels were prepared with a four-arm PEG-Ac macromer and PEG-diSH crosslinker at pH 7.4. This experiment also established a functional upper limit of the gels’ concentration and number of arms. PEG macromers with eight arms at a concentration of 30% formed too rapidly to reliably inject, even when those injections were performed immediately. 

We also tested the effect of pH on hydrogels made with an eight-arm PEG-Ac macromer and PEG-diSH crosslinker at a 20% *w*/*v* concentration (Figure 3E,F). We found that hydrogels formed at a low pH (6.8) degraded faster than hydrogels formed at higher pH values (7.4). Despite the strong effect of pH on gelation time, we observed that a decrease in pH had less of an effect on the gel longevity than the polymer concentration or the number of macromer arms. 

All of the hydrogels described above were degradable via ester hydrolysis, and some of them degraded faster in vivo than expected from prior in vitro experiments [19,20]. To slow in vivo degradation, we next tested various combinations of degradable and non-degradable PEG macromers and crosslinkers (Figure 3G,H). Hydrogels were made at a 10% *w*/*v* polymer concentration and a pH of 7.4. We made degradable hydrogels by forming a thioester bond by using either an eight-arm PEG-Ac macromer (Ac, degradable) and a PEG-diSH crosslinker (diSH, non-degradable) or an eight-arm PEG-VS macromer (VS, non-degradable) and a PEG-DD2 crosslinker (DD2, degradable). By 48 h, the gels made with VS + DD2 were completely absent, while gels made with Ac + diSH were still present, albeit had decreased in size. As expected, the gels formed from non-degradable polymers (VS + diSH) did not show signs of degradation over the time of the experiment. Likewise, gels made with a combination of non-degradable and degradable crosslinkers degraded noticeably slower.

### 2.3. Delayed Release of PEG-Encapsulated rhGALNS In Vitro

**Figure 4:**
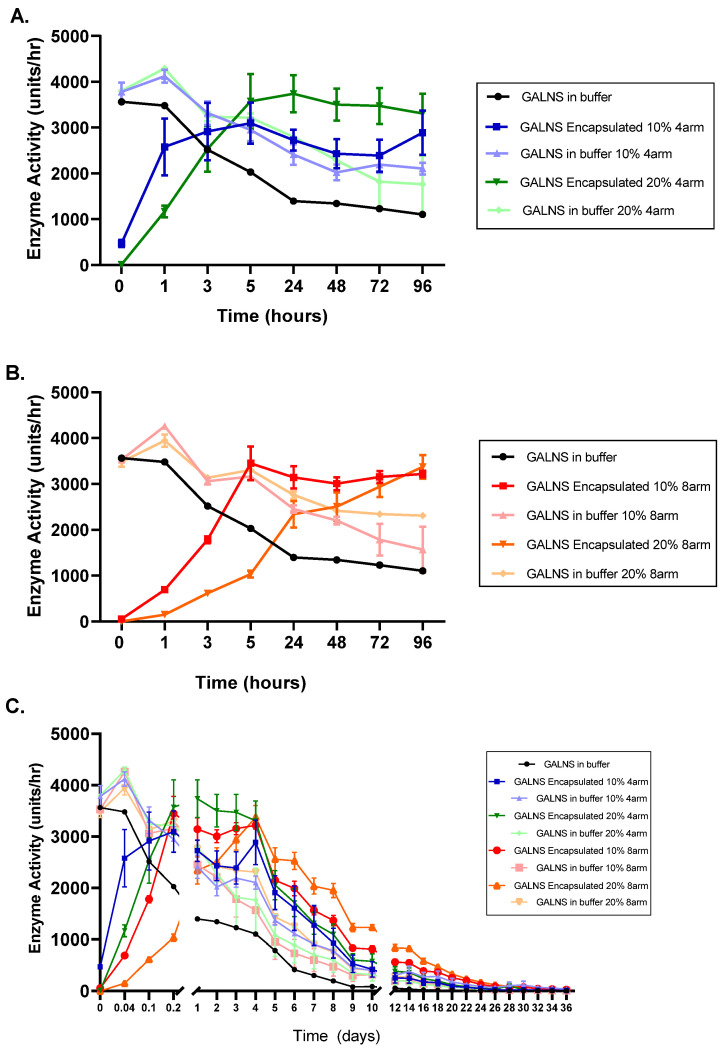
Enzyme activity as a function of hydrogel type. The rhGALNS enzyme was encapsulated in PEG hydrogels formed with (**A**) four-arm PEG-Ac or (**B**) eight-arm PEG-Ac and a PEG-diSH crosslinker at a buffer pH of 7.4. Figures (**A**,**B**) show, in detail, the first 96 h of rhGALNS release into the buffer. The hydrogels were 10% and 20% *w*/*v* in total polymer concentration. rhGALNS was either encapsulated in the hydrogel, with its activity measured upon release, or simply incubated in buffer in the presence of the hydrogel. (**C**) GALNS enzyme activity was followed up to 36 days under the same conditions outlined in (**A**,**B**).

We next tested the ability of hydrogels to continually release active encapsulated rhGALNS in vitro. We formed PEG hydrogels with the encapsulated active rhGALNS enzyme and incubated the hydrogels in release buffer at 37 °C. Starting immediately after gel formation (t = 0) and ending at 96 h post-gelation, we periodically collected release buffer samples and measured the enzyme activity of these releasates (Figure 4). As controls, we compared the activity of rhGALNS in buffer only or rhGALNS incubated in release buffer that also contained a PEG gel (rhGALNS not encapsulated in the gel) to determine whether the gel itself had some effect on the measured enzyme activity. We used gels made at pH 7.4 and with four-arm or eight-arm PEG-Ac and PEG-diSH crosslinkers at 10% or 20% *w*/*v*. For rhGALNS in solution, we noted a quick and steady decrease in enzyme activity over time, as expected. Interestingly, the activity of rhGALNS incubated in buffer that also contained PEG gels was higher than that in buffer that did not contain gels. After an initial burst release, the enzyme activity (i.e., active enzyme released) reached a plateau at 3 h (fastest release) for the 10% *w*/*v* and at 5 h for the 20% *w*/*v* gels made with four-arm PEG-Ac (Figure 4A). A plateau in enzyme activity was reached at 5 h for the 10% *w*/*v* and at 96 h for the 20% *w*/*v* (slowest release) for the gels made with eight-arm PEG-Ac (Figure 4B). By ~5–24 h, the enzyme activity was higher for encapsulated rhGALNS than for rhGALNS in solution for all conditions. At the end of the 96-h period, we continued the regular collection of the release buffer from the hydrogels. We observed that the non-encapsulated rhGALNS enzyme showed no activity after 9 days. By contrast, we found that hydrogels continued to release active rhGALNS for much longer, with 20% *w*/*v* eight-arm PEG-Ac hydrogels continuing to show active GALNS in its releasate for up to 28 days from the initial hydrogel encapsulation (Figure 4C).

### 2.4. No Change in the Free Energy of the Unfolding of rhGALNS in the Presence of PEG

**Figure 5:**
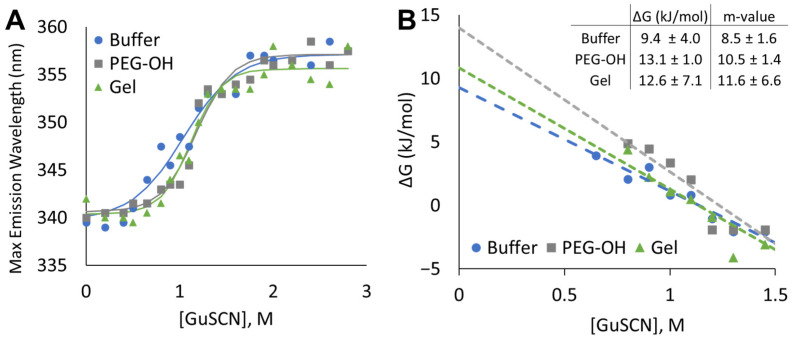
Free energy of unfolding for rhGALNS in different environments upon exposure to the denaturant. Maximum emission intensity wavelength, Ex: 285 nm and Em: 320–380 nm, (**A**) and free energy (**B**) for rhGALNS (25 µg/mL) in the buffer control, PEG-OH solution, and when encapsulated in a 15% *w*/*v* hydrogel prepared from a four-arm PEG-AC macromer and PEG-diSH crosslinker at a buffer pH of 7.4. *n* = 3. Dashed lines represent linear extrapolation to zero denaturant concentration. R^2^ of 0.94, 0.88, and 0.88 for *n* = 4, 3, and 4 of Buffer, PEG-OH, and Gel, respectively.

The stability of the tertiary structure of rhGALNS was quantified by the free energy of unfolding (ΔG) measured via intrinsic fluorescence. rhGALNS was encapsulated in 15% *w*/*v* four-arm PEG-Ac + PEG-diSH (Gel) and also dissolved in a solution containing 15% *w*/*v* uncrosslinked PEG polymer (PEG-OH) and exposed to 0–3 M GuSCN denaturant (Figure 5A). rhGALNS in HEPES buffer (Buffer) served as a control. Unfolding was monitored via a shift in the maximum emission wavelength (*λ*_max_) from approximately 340 nm (native) to 355 nm (unfolded), with intermediate wavelengths representing an equilibrium between the two states per the two-state unfolding model. The free energy in each denaturant concentration was calculated from the equilibration constant, and the linear portion was extrapolated to zero denaturant concentration to determine the free energy of unfolding (Figure 5B). The slope of the regression (*m*-value) indicates the increase in the surface area of rhGALNS upon unfolding. The ΔG of PEG-containing samples trended higher than that of the buffer control, although the effect was not statistically significant. If crowding with PEG-OH or confinement within the PEG hydrogel had a significant stabilizing effect on rhGALNS, we would expect a more compact unfolded conformation and a lower *m*-value [21]. However, we observed no statistical difference in the *m*-value between Gel, PEG-OH, and Buffer, suggesting that the gel did not affect the stability of the rhGALNS tertiary structure upon encapsulation.

### 2.5. rhGALNS Conjugates with PEG-Acrylates at Long Reaction Times 

**Figure 6:**
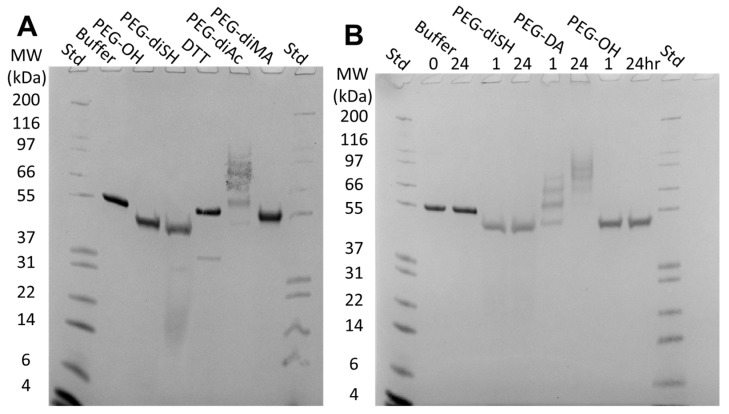
(**A**) Non-reduced SDS-PAGE of rhGALNS (40 µg/mL) after 1 h of incubation with various PEG polymers (10% *w*/*v*) or DTT (0.15% *w*/*v*). All samples were run non-reduced. (**B**) Non-reduced SDS-PAGE of rhGALNS (40 µg/mL) after incubation with PEG-diSH, PEG-DA, or PEG-OH (10% *w*/*v*) for 1 and 24 h. All incubations were performed at room temperature.

To determine if the PEG polymer reactive groups react with rhGALNS, we incubated rhGALNS with two unsaturated carbonyls, PEG-DA (5 kDa, 10% *w*/*v* in HEPES buffer, 40 mM reactive groups) and PEG-DM (1 kDa, 10% *w*/*v* in HEPES buffer, 20 mM reactive groups), and two thiol crosslinkers, PEG-diSH (10 kDa, 10% *w*/*v* in HEPES buffer, 20 mM reactive groups) and DTT (0.15% *w*/*v* in HEPES buffer, 20 mM reactive groups). rhGALNS incubated in HEPES buffer and 10% *w*/*v* PEG-OH (6 kDa) served as controls. The samples were incubated for 1 h at room temperature and analyzed by non-reducing sodium dodecyl sulfate-polyacrylamide gel electrophoresis (SDS-PAGE) (Figure 6A). As we have observed previously, the presence of PEG altered the lane migration in SDS-PAGE, and the molecular weight ladder could not be directly correlated to bands in PEG-containing samples [22]. Faint low-molecular-weight bands were observed in both PEG-diSH and DTT, indicating a potential reduction in disulfide bonds and the liberation of cleaved fragments. PEG-DM had no apparent effect, but PEG-DA formed high-molecular-weight bands. PEG-diSH and PEG-DA were also analyzed up to 24 h of incubation (Figure 6B). The high-molecular-weight bands of PEG-DA increased with the incubation time, indicating a slow conjugation reaction between the acrylate groups and rhGALNS.

### 2.6. Encapsulation and Release Did Not Have an Impact on the rhGALNS Secondary Structure 

**Figure 7:**
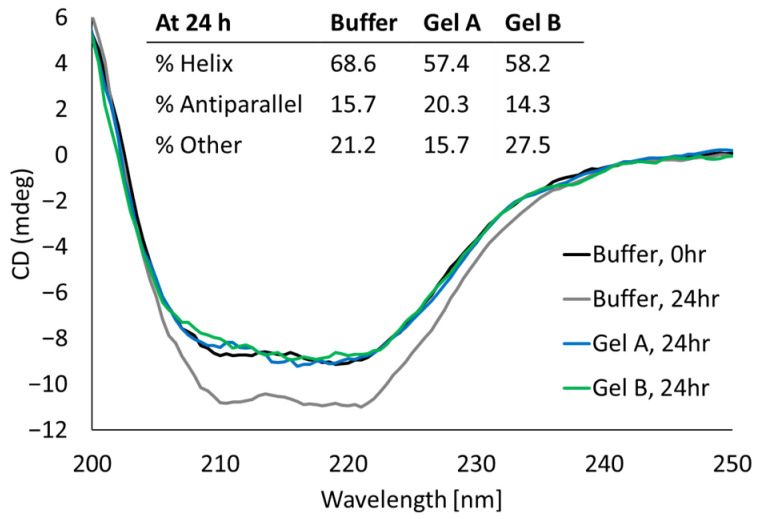
Circular dichroism for rhGALNS. The following rhGALNS samples were prepared and measured: 0.1 mg/mL rhGALNS stock solution (Buffer, 0 h), rhGALNS incubated at 37 °C for 24 h in buffer (Buffer, 24 h), and rhGALNS incubated at 37 °C for 24 h in two gel types, namely, loaded by direct encapsulation during gelation (Gel A, 24 h) and by swelling a dried, blank gel in rhGALNS solution (Gel B, 24 h). Measurements of rhGALNS were taken upon release from the gels. Spectra were blanked against either the release buffer or the releasate of a corresponding blank gel. Ratio of rhGALNS secondary structures was calculated using the BeStSel online CD spectra analyzing tool.

To determine if encapsulation in the PEG hydrogels or release from the PEG hydrogels had an impact on the enzyme secondary structure, we compared the circular dichroism (CD) spectra of rhGALNS released from 15% *w*/*v* four-arm PEG-Ac + PEG-diSH hydrogels to those of rhGALNS in buffer (Figure 7). GALNS was loaded into the gel both by direct encapsulation during gelation (Gel A) and by swelling a dried, blank gel in rhGALNS solution (Gel B). Measurements were taken in the release buffer at pH 5.5, which more closely represents the acidic environment that native GALNS would experience within the lysosome. The spectra were comparable between all samples, indicating no major shift in the secondary structure from either method of gel loading. The ratios of secondary structures showed little change as a function of hydrogel encapsulation either during or post-gelation. 

### 2.7. In Vivo Sustained Release from Hydrogel-Encapsulated Proteins in C57BL/6 Albino Mice

**Figure 8:**
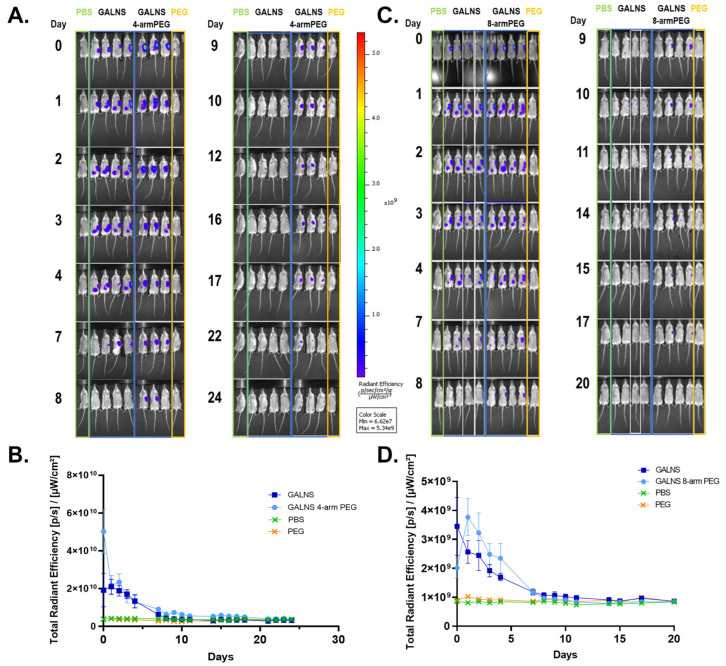
Representative whole body in vivo fluorescence images of live C57BL/6 albino mice with (**A**,**B**) non-encapsulated (*n* = 4) or encapsulated (*n* = 3) Alexa 680-labeled rhGALNS in four-arm PEG-Ac + PEG-diSH hydrogels. Images were taken daily until 24 days post-sub-Q injections of rhGALNS at a dose of 5 mg/kg body weight. (**C**,**D**) Non-encapsulated (*n* = 4) or encapsulated (*n* = 4) Alexa 680-labeled-rhGALNS in eight-arm PEG-Ac + PEG-diSH hydrogels. Images were taken daily until 20 days post-sub-Q injections of rhGALNS at a dose of 5 mg/kg body weight. (**B**,**D**) Quantitative analysis of the IVIS images. PBS: Mice injected with PBS; PEG: Mice injected with the PEG hydrogel. Rainbow images show the relative levels of fluorescence ranging from low (blue) to high (yellow/red). Values are expressed as the mean ± SEM (*n* = 4).

To demonstrate the effect of the hydrogels in stabilizing rhGALNS and facilitating a sustained release, we looked at the hydrogel-encapsulated enzyme in vivo. First, we used Bovine Serum Albumin (BSA) with a molecular weight of 66.5 KDa as a model stable protein. Alexa 680-labeled BSA (10 mg/kg of body weight) was encapsulated in four-arm PEG-Ac hydrogels, and its fluorescence was compared to that of control non-encapsulated BSA. We found that there was a significant difference in intensity after 24 h of subcutaneous injection into the left flank of mice. Non-encapsulated BSA was eliminated from the mice within 24 h, while the signal of the BSA encapsulated in the PEG hydrogel was present until 10 days post-injection (Figure S2). Maintaining the same hydrogel formulation (20% *w*/*v* four-arm PEG-Ac + PEG-diSH), we injected Alexa 680-labeled rhGALNS at a concentration of 5 mg/kg of body weight. We found that non-encapsulated rhGALNS was eliminated from the body at 7 days post-injection, while hydrogel-encapsulated rhGALNS was present until 23 days post-injection (Figure 8A,B). To evaluate differences between four-arm PEG-Ac and eight-arm PEG-Ac, we used a hydrogel formulation of 20% *w*/*v* eight-arm PEG-Ac + PEG-diSH to encapsulate 5 mg/kg of body weight of Alexa 680-labeled rhGALNS. Likewise, we found that the non-encapsulated rhGALNS was eliminated at 7 days post-injection, while the rhGALNS encapsulated in the eight-arm PEG-Ac + PEG-diSH gels was detected until 17 days post-injection (Figure 8C,D), highlighting the importance of the sustained release hydrogel device.

## 3. Discussion

The overall goal of our work is to develop an injectable PEG hydrogel delivery system that can encapsulate rhGALNS, retain its activity, and release it in a sustained manner over 7–28 days. While we have optimized degradable hydrogels for drug delivery in previous works, this approach has not been studied for the delivery of enzyme replacement therapies [19,20]. At a minimum, an injectable PEG hydrogel would improve patient quality of life by reducing administration from multi-hour infusion to a subcutaneous injection that takes several minutes. Poor biodistribution is a common limitation of ERT for lysosomal storage diseases due to the cellular uptake of the enzyme being limited by receptor-mediated pathways [23]. The majority of the enzyme administered by infusion is either cleared or inactivated, and the remaining amount of rhGALNS taken into cells is retained for up to seven days [24], thus requiring weekly infusions. We expect that a sustained release profile from a hydrogel would be a more efficient treatment in terms of the total enzyme administered. For example, a hydrogel designed to release an enzyme over seven days will result in retained activity for up to 14 days, decreasing the need for patient visits by half. Further, the production and purification of recombinant proteins are expensive, and a more protein-efficient delivery of rhGALNS could significantly reduce the prohibitive treatment cost [10,25].

We chose PEG hydrogels due to their excellent biocompatibility and tunability [12,13]. Michael-type addition is suitable for enzyme encapsulation due to the highly specific nature of the reaction chemistry and the water-based gelation mechanism, which allow for enzyme encapsulation directly in the hydrogel precursor solution, which could then be injected for in vivo gelation [26,27]. Although the thiol-acrylate chemistry can result in some PEGylation of the enzyme payload and a reduction in enzyme activity [28,29], we have observed a minimal impact on enzymes in the fast-forming gels described here [22]. Further, the gelation reaction results in hydrolytically degradable thioester crosslinks, providing a means of in vivo biodegradation and eliminating the need for the surgical removal of the gel. The reaction also allows for a tunable gelation time. When preparing gels for subcutaneous injection, the gelation time must be balanced between a delayed gelation to allow for time for injection and a rapid gelation to prevent the dilution of the gel precursor solution post-injection. The gelation time may be tuned to the specific need via the reaction pH, the PEG concentration, and the choice of crosslinking reaction groups (Figure 2). However, these conditions must be chosen with the limitation of the payload enzyme in mind. Although pH 7.4 was chosen for the in vitro and in vivo release experiments in this study due to its ideal gelation time, a high pH is detrimental to rhGALNS activity, a lysosomal enzyme, and may not be the ideal pH choice [19].

We also examined the effect of the reaction pH, the PEG concentration, and the choice of crosslinking reaction groups on the degradation time in vivo. The hydrolytic degradation of Michael-type addition thioester bonds both drives protein release from the gel and allows the gel to be cleared from the body. Corroborating our prior in vitro work [19], we observed that conditions resulting in fast gelation times also increased degradation times. The ester at each crosslink is the primary point of bond cleavage in the mesh network, and each is assumed to be equally susceptible to hydrolysis. Such degradation can be modeled through random scission of the polymer network, where the degradation kinetics are proportional to the crosslinking density [30]. Thus, as expected, we observed that high polymer concentrations and multi-armed PEGs led to an increased degradation time due to the higher crosslink density. The degradation rate may also be tuned through crosslinker chemistry, either by adding nondegradable crosslinks (e.g., 1:1 diSH/DD2) to improve the polymer network integrity or by modifying the hydrophobicity, steric hindrance, or electronegativity of moieties near the ester to modulate its susceptibility to hydrolysis [20]. However, in our previous work, we found that acrylate/thiol PEG hydrogels degraded in vitro in ~16 days [20], but similar gels tested in vivo degraded in <48 h. This accelerated rate may be linked to a lower crosslinking density due to in vivo gelation upon injection, as the precursor solution spreads throughout the tissue and could mix with interstitial fluid, resulting in a gel with a lower crosslink density. The movement of the mouse may also contribute to faster degradation times, as cyclic compression has been shown to accelerate the biodegradation of hydrolytically labile gels in vitro [31]. Additionally, in vitro degradation studies are typically performed using gels with consistent, regular geometry, while the gels extracted from our mice were found in a variety of shapes as they took the space available to them upon injection (Figure 4). These irregular shapes may fragment and accelerate the degradation and clearance of the gel. The irregular shapes may result in a faster release of rhGALNS from gels in vivo than expected from the in vitro models. To address consistency, future work will compare injectable hydrogel formulations to injectable pre-formed hydrogel microspheres as well as implantable pre-formed hydrogel slabs.

Our in vitro release data demonstrated that the delayed release of encapsulated rhGALNS preserved activity over the buffer for up to 28 days (Figure 4). This supports our target release window of 7 or more days. However, the presence of “blank” PEG gels in a solution of rhGALNS also preserved activity better than the buffer alone. This suggests that the stabilization of rhGALNS may not be from encapsulation alone but rather an interaction between rhGALNS and the PEG backbone. PEG has been shown to increase the activity of some enzymes beyond what is expected from volume-exclusion alone [32]. Although confinement is predicted to stabilize compact, globular enzymes such as rhGALNS, this has not been universally observed [33,34]. An analysis of the tertiary structure of encapsulated rhGALNS via intrinsic fluorescence did not contradict this hypothesis. The free energy of the unfolding of rhGALNS trended higher when confined within the hydrogel and when in a solution with uncrosslinked PEG, although the effect was not significant (Figure 5). Likewise, the *m*-value, an indication of the change in the surface area between the folded and unfolded states [21], was similar between the PEG gel, PEG solution, and buffer. If confinement was the driver for rhGALNS stabilization, we would expect to see a lower *m*-value in the gel, as the hydrogel mesh would restrict conformations with a large surface area and encourage a more compact unfolded state [33]. The gels used in this analysis, namely, 15% *w*/*v* four-arm PEG-Ac (10 kDa) + PEG-diSH (3.4 kDa), have a mesh size of 8.5 nm, or approximately 2–3 times the hydrodynamic radius of the rhGALNS monomer (Table 1). The effect of confinement is expected to increase as the dimensions of the confining environment approach the protein hydrodynamic radius [33,35]. 

Likewise, the secondary structure of released rhGALNS was not significantly altered by encapsulation (Figure 7). As other proteins have been shown to be susceptible to PEGylation when encapsulated prior to hydrogel crosslinking, we compared two loading strategies for the gels [28]. First, we used direct encapsulation during gelation, where rhGALNS was exposed to high concentrations of reactive PEG polymers (PEG-acrylates and PEG-thiols) in the precursor solution. Second, we loaded the enzyme by swelling a pre-formed gel in rhGALNS solution, where rhGALNS was only exposed to residual reactive PEG polymers. In both cases, the secondary structure of the released rhGALNS was comparable to that of the rhGALNS prior to gel loading. This suggests that the PEGylation reactions have no practical negative impact on the structure of the rhGALNS that is released from the gel. However, PEGylated rhGALNS may not be fully represented in the fractions used for CD, as PEGylated rhGALNS would likely be covalently linked to the hydrogel matrix and have a delayed release. Although we have demonstrated via SDS-PAGE that the rhGALNS reacts with PEG-acrylates to form high-molecular-weight PEGylated products, those incubations were for at least 1 h in PEG-acrylate without the addition of a crosslinker (Figure 6). In gels prepared for subcutaneous injection, the polymers and rhGALNS solutions are mixed rapidly prior to use. The concentration of reactive PEG-acrylate drops rapidly as gelation occurs in less than 30 min. Thus, rhGALNS is exposed to lower concentrations of PEG-acrylate for shorter times when encapsulated in PEG gels compared to the SDS-PAGE experiment.

To confirm our release window in vivo, we used the optimal in vitro hydrogel formulations (20% *w*/*v* four-arm and eight-arm PEG-AC + PEG-diSH; optimized for prolonged sustained release and prolonged degradation time) to encapsulate fluorescently labeled proteins (e.g., BSA, rhGALNS). When not encapsulated, rhGALNS was cleared in 7 days, which is consistent with a study that suggests that GALNS is retained intracellularly for 7 days [24]. On the other hand, encapsulated rhGALNS remained in mice between 17 and 20 days post-injection (Figure 8A,C). The images suggest that rhGALNS is present at the injection site as well as the liver and spleen, two key organs for enzyme uptake in lysosomal storage diseases, which is a promising sign suggestive of sustained efficacy [23]. In future studies, fluorescently labeled PEG may be used to track the hydrogel degradation time and map the clearance of the PEG more accurately. 

The data presented here are a promising indication that the sustained release of rhGALNS from a PEG hydrogel is a feasible alternative to i.v. infusion. However, further work is needed to overcome the challenges of this mode of administration and study its efficacy in more detail. For example, the time-sensitivity of the gelation, the instability of rhGALNS, and the hydrolytic degradation of the gel pose a logistical challenge for the production and shipment of the gel. Thus, as an alternative to in situ gelation, we plan to investigate microspheres that may be stored and shipped in a lyophilized state. Any technology that negates the need for long infusions could be beneficial for children who currently miss one day of school per week due to the need for having infusions administered at a medical center. Further, while our studies are focused on improving the quality of life of Morquio A patients, a delivery system such as the one described here could be expanded to other lysosomal storage diseases that are treated through ERT. 

## 4. Materials and Methods

### 4.1. Reagents

Polyethylene glycol (PEG-OH; 6 kDa) was obtained from Alfa Aesar (Haverhill, MA, USA). PEG-diacrylate (PEG-DA; 5 kDa), PEG-dimethacrylate (PEG-DM; 1 kDa), four-arm polyethylene glycol-acrylate (four-arm PEG-Ac; 10 kDa), eight-arm polyethylene glycol-acrylate (eight-arm PEG-Ac; 20 kDa), four-arm polyethylene glycol-vinylsulfone (four-arm PEG-VS, 10 kDa), eight-arm polyethylene glycol-vinylsulfone (eight-arm PEG-VS; 20 kDa), and polyethylene glycol-dithiol (PEG-diSH; 3.4 kDa) were purchased from Laysan Bio Inc. (Arab, AL, USA). The polyethylene glycol diester-dithiol (PEG-DD2) crosslinker was synthesized in-house as previously described [19,20]. HEPES, CM-sepharose CL-6B resin, Medium Essential Medium (MEM), Dulbecco’s Modified Eagles Medium (DMEM), Fetal Bovine Serum (FBS), Geneticin (G418), proline, sodium acetate, ß-glycerophosphate, penicillin/streptomycin, sodium carbonate, Folin-Ciocalteau’s phenol reagent, and sodium dideoxycholate were purchased from Sigma Aldrich (St. Louis, MO, USA). Sephacryl S-100 HR gel filtration resin was purchased from GE Healthcare Life Sciences (Pittsburgh, PA, USA). Four-methylumbelliferyl (4MU)-β-D-galactopyranoside-6-sulfate was purchased from Moscerdam Substrate (Rotterdam, The Netherlands). Chinese Hamster Ovary (CHO) cells were purchased from the American Type Culture Collection (Manassas, VA, USA). EX-Cell Collection media were purchased from Gibco (Amarillo, TX, USA). NuPAGE, Novex, and SimplyBlue Safestain and Pierce In-Solution Tryptic Digestion and Guanidination were from Thermo Fisher Scientific (Waltham, MA, USA). Barium chloride, iodine, and perchloric acid were from Fisher Chemicals (Hampton, NH, USA). Guanidine thiocyanate (GuSCN) was from Promega (Madison, WI, USA). Dithiothreitol (DTT) was from Millipore Sigma (Burlington, MA, USA).

### 4.2. Cell Culture and rhGALNS Purification

CHO cells were transfected to constitutively express rhGALNS, as described previously [4]. Cells were cultured in DMEM with 15% FBS prior to enzyme collection. Once the cells reached confluence, the media were switched to EX-cell collection media for conditioning and collected every 24 h. rhGALNS was isolated from conditioned EX-cell media as described previously [4]. Briefly, following collection, the media were sterile-filtered. The conditioned media were then concentrated and dialyzed using a buffer of 25 mM sodium acetate and 1 mM β-glycerophosphate at pH 5.5. Next, the concentrated media were loaded onto a CM Sepharose resin (GE), followed by a size-exclusion separation using an S-100 resin (GE). rhGALNS-containing fractions were pooled, concentrated, and stored at −80 °C until use. 

### 4.3. Assessment of rhGALNS Activity

rhGALNS activity was assayed as described previously [4,19]. Briefly, releasate was incubated with the 22 mM 4-MU-β-D-galactopyranoside-6-sulfate substrate for 15 min at 37 °C and then incubated for 30 min with 10 mM β-galactosidase before measurement by fluorometry (Excitation of 365 nm, Emission of 445 nm) [36]. 

### 4.4. Hydrogel Preparation

PEG polymers were dissolved separately in 0.1 M HEPES buffer at pH values ranging from 6.5 to 7.4, in concentrations ranging from 10% to 30% *w*/*v*. Polymer solutions were mixed in a 1:1 molar ratio between the Michael-Type addition donor groups of the crosslinkers (PEG-diSH or PEG-DD2) and the acceptor groups of the multi-arm PEG macromers (four-arm PEG-Ac, eight-arm PEG-Ac, four-arm PEG-VS, or eight-arm PEG-VS). The mixtures were left to initiate gelation within the syringe and injected immediately prior to fully solidifying. 

### 4.5. Animals

C57BL/6J mice (4 weeks old) or C57BL/6 albino mice (7 weeks old) were selected for all the experiments in this study. All mice were housed in a pathogen-free environment with a normal diet. All procedures were in accordance with the Institutional Animal Care and Use Committee (IACUC) guidelines under approved protocols at Saint Louis University and followed the NIH’s Guide for the Care and Use of Laboratory Animals [37].

### 4.6. In Vivo Gelation Studies

Subcutaneous injections of hydrogels were performed on 4-week-old mice. rhGALNS-free hydrogel mixtures were prepared as described above. Prior to fully solidifying, hydrogel mixtures were subcutaneously injected into the left and right flack of each mouse. Mice were sacrificed after 24, 48, 72, and 96 h post-injection, and the remaining gels were removed for a size comparison.

### 4.7. Encapsulation of rhGALNS 

rhGALNS was encapsulated into the gels by mixing 4000 units of the rhGALNS enzyme in the hydrogel macromer solution prior to combining with the crosslinker solution, as described above. Gels were formed into uniformly shaped slabs by pipetting 30 µL of the mixture onto a parafilm-coated glass plate and covering the gel with another parafilm-coated plate 1 mm apart (separated by silicon spacers).

### 4.8. Measurement of rhGALNS Bulk Release from Hydrogels

Following encapsulation, hydrogels were incubated in 270 µL of rhGALNS release buffer (25 mM sodium acetate, 1 mM β-glycerophosphate pH 5.5, 100 mM NaCl) and incubated at 37 °C with shaking. Sampling was performed by removing 30 µL of the release buffer and replacing it with an equal volume of fresh buffer. Immediately after collection, rhGALNS activity was assayed for these samples. Initial collections were performed at the start of the experiment, as well as at 1, 3, and 5 h following incubation. Further sample collection occurred every 24 h, which was extended to every 48 h after 10 days of incubation. To assess the preservation of enzyme activity, hydrogels were prepared for each time point (a separate hydrogel for each time point) as mentioned above, and the entire releasate sample was removed. The releasate samples were immediately placed at −80 °C for storage until testing or used immediately for further analysis of enzyme activity, as described previously.

### 4.9. Measurement of Hydrogel Gelation Time 

Hydrogel gelation time was measured by the inverted tube method [38]. Briefly, 50 µL of the hydrogel precursor solution was pipetted into a microfuge tube and inverted constantly. The gelation time was noted when the gel precursor solution stopped flowing. When noted, the precursor solution was extruded through a 30-gauge syringe needle into the microcentrifuge tube rather than pipetting to simulate a sub-cutaneous injection.

### 4.10. Free Energy of Unfolding

To measure the free energy of unfolding, hydrogels were prepared as described above with some minor modifications. Briefly, stock solutions of rhGALNS (0.5 mg/mL), four-arm PEG-Ac (20% *w*/*v*), and PEG-diSH (20% *w*/*v*) were prepared in 0.1 M HEPES, pH 7.4. The stock solutions were then used to prepare a hydrogel precursor solution of 50 µg/mL rhGALNS and 15% *w*/*v* PEG with stoichiometrically equal acrylate and thiol groups. The hydrogel precursor solution was pipetted into UV-star clear bottom 96-well plates at 70 µL per well. After 60 min of gelation, 95 µL of the denaturant (GuSCN in 0.1 M HEPES (pH 7.4, 0–3 M) was added to each well and mixed on a Fisher orbital shaker (Waltham, MA, USA) at 200 rpm for 2 h. Then, the intrinsic fluorescence intensity (Excitation of 285 nm, Emission of 320–380 nm, 1 nm step) of rhGALNS in the presence of the denaturant was measured on a Spectra Max M5 with SoftMax Pro Software (Molecular Devices, San Jose, CA, USA). The energy of unfolding was calculated from solvent denaturation curves using the linear extrapolation method [39,40].

### 4.11. Non-Reducing SDS-PAGE 

rhGALNS at 40 µg/mL was incubated in HEPES buffer (control), 0.15% *w*/*v* DTT, and 10% *w*/*v* PEG solutions: PEG-DA (5 kDa), PEG-diSH (10 kDa), PEG-DM (1 kDa), and PEG-OH (6 kDa) for up to 24 h at room temperature. After incubation, the samples were prepared according to the manufacturer’s protocols for non-reduced SDS-PAGE. PAGE gels were run at a constant 200 V for 40 min on a Bis-Tris 4–12% gradient gel in an XCell SureLock cell (Thermo Fisher, Waltham, MA, USA). The lanes were loaded with a sample volume corresponding to 0.4 µg of rhGALNS. The gels were stained with SimplyBlue Safestain.

### 4.12. Circular Dichroism (CD)

The secondary structure of rhGALNS released from PEG hydrogels was measured using a JASCO 715 Circular Dichroism Spectrophotometer (JASCO Applied Sciences, Victoria, BC, Canada). Briefly, rhGALNS was concentrated or diluted with release buffer to 0.1 mg/mL prior to measurements. Far UV spectra (190–260 nm) were obtained with a 1.0 nm bandwidth, 0.5 nm step interval, and 1.5 s/nm scanning speed. The secondary structure was calculated using the BeStSel online CD spectra analyzing tool [41].

### 4.13. In Vivo Fluorescence Imaging and Image Analysis

First, 7-week-old male C57BL/6 albino mice received a single subcutaneous injection of fluorescently labeled BSA or rhGALNS. The proteins were labeled with ALEXA 680 (ThermoFisher Scientific, Waltham, MA, USA) according to the manufacturer’s instructions. Precursor solutions were prepared to reach a final concentration of 20% *w*/*v* four-arm PEG-Ac or eight-arm PEG-Ac + PEG-diSH, loaded with BSA or rhGALNS. Hydrogel mixtures (200 µL) were subcutaneously injected into the left flank of each mouse. The mice were anesthetized with isoflurane, and fluorescent images were acquired on an IVIS Spectrum imager (Perkin Elmer, Hongkong, China) every 24 h for up to 24 days.

All IVIS images were acquired under the same field of view using autoexposure. The data were analyzed using Living Image software (version 4.5). Fluorescent regions were quantified and expressed as Radiant Efficiency (p/s/sr)/(µW/cm^2^). Analyses were conducted with a Holm–Šídák’s multiple comparison test. *p* < 0.05 was considered statistically significant.

### 4.14. Mesh Size Determination

The mesh size (*ξ*) of the hydrogels was determined according to the Flory–Rehner theory, as described previously [20,22]. In brief, the hydrogel mass was measured immediately after fabrication (M_G_), after swelling in HEPES buffer at pH 7.4 for 24 h (M_S_), and after drying in a 60 °C oven overnight (M_D_). The swelling ratio, Q_m_, which is the ratio of swollen mass to dry mass (M_S_/M_D_), was then used to calculate the mesh size per Equation (1) [42].

(1) $$\xi ={\left(\upsilon_{2,s}\right)}^{-\frac{1}{3}}{\left(\frac{2C_{n}\bar{M_{c}}}{M_{r}}\right)}^{\frac{1}{2}}l$$

where υ_2,*s*_ is the polymer volume fraction in the swollen state (calculated from Q_m_), *Cn* is the characteristic ratio for PEG, *M_C_* is the average molecular weight between two adjacent crosslinks, *M_r_* is the molecular weight of a PEG repeat unit, and l is the average bond length between the C-C and C-O bonds in a PEG repeat unit.

### 4.15. Statistical Analysis

All experiments were conducted at least in triplicate, and the results are expressed as an average ± standard deviation. On average, three to six samples were used for each independent experiment. Analyses were performed with IBM SPSS Statistics 23.0 software. One-way ANOVA, along with Tukey’s post hoc test, was used to assess significance within groups. A two-tailed paired *t*-test was used to compare the differences between two groups. *p* < 0.05 was considered statistically significant.

## 5. Conclusions

In this study, we investigated the suitability of PEG hydrogels as a potential rhGALNS sustained delivery system for the potential treatment of Morquio A syndrome. Gels formed with a low pH, high PEG concentration, and high number of PEG arms were found to have the fastest gelation times, but certain combinations of these factors gelled too quickly (in seconds) to be feasible for subcutaneous injection. A high PEG concentration and a high number of PEG arms also resulted in the longest degradation times due to their higher crosslink density. However, we observed degradation rates that were faster than our previous in vitro studies and hypothesize that a low crosslink density, irregular gelation, and mechanical stimulation in vivo may be accelerating the breakdown of the gel. We encapsulated rhGALNS in the PEG hydrogels and observed a retention in enzymatic activity relative to the buffer for up to 28 days. However, the presence of blank PEG gels alongside unencapsulated rhGALNS also preserved activity, suggesting that stabilization may result from the interaction between rhGALNS and the PEG backbone rather than encapsulation shielding the protein. Likewise, the encapsulation of rhGALNS did not significantly alter its free energy of unfolding compared to the buffer or solutions of an uncrosslinked PEG polymer. While a PEGylation reaction was observed between PEG-acrylate and rhGALNS, there was no change in the secondary structure of the released rhGALNS, as measured by CD. Lastly, we administered fluorescently labeled rhGALNS to mice via subcutaneous injection and observed the presence of rhGALNS for up to 20 days when delivered in a hydrogel, compared to 7 days for rhGALNS in the buffer solution. Together, these results indicate that PEG hydrogels are a suitable drug delivery system for preserving and releasing active rhGALNS. The tunability of the Michael-type addition gelation reaction allows for a formulation tailored for both rhGALNS activity and a gelation time optimized for subcutaneous injection. The PEG hydrogel preserves and releases active rhGALNS over a time window significantly longer than that of the current standard treatment, although future studies are needed to compare the efficacy of the treatments.

## 6. Patents

A.M.M. and S.P.Z. are inventors on the U.S. Patent No. 11,034,950 related to this work.

## Figures and Tables

**Table 1:** Swelling ratio and mesh size for PEG hydrogels.

Gel Types	[PEG] (%*w*/*v*)	Mesh Size (nm)	Swelling Ratio, Qm
Four-Arm Acrylate, 10 kDa + PEG diSH, 3.4 kDa	10	9.4 ± 1.5	16.1 ± 3.8
	15	8.5 ± 0.2	13.2 ± 0.6
	20	8.5 ± 0.4	13.1 ± 0.7
Eight-Arm Acrylate, 20 kDa + PEG diSH, 3.4 kDa	10	9.3 ± 0.7	15.3 ± 1.6
	15	9.2 ± 1.4	13.6 ± 1.7
	20	7.4 ± 0.6	10.4 ± 0.7

## Data Availability

Data is contained within the article.

## Supplementary Materials

The following supporting information can be downloaded at: https://www.mdpi.com/article/10.3390/ph16070931/s1, Figure S1: Effect of pH, polymer concentration, and macromer: crosslinker on gelation time after mock subcutaneous injection; Figure S2: Representative whole body in vivo fluorescence images of live C57BL/6 albino mice with non-encapsulated or encapsulated labeled-BSA.
