# Supplementary material for: Hydrogel Delivery Device for the In Vitro and In Vivo Sustained Release of Active rhGALNS Enzyme
Source: Pharmaceuticals (Basel). 2023 Jun 27;16(7):931. doi: 10.3390/ph16070931 (PMC10384033; doi:10.3390/ph16070931)
Supplement: Supplementary file 1 [file pharmaceuticals-16-00931-s001.zip › pharmaceuticals-2410269-supplementary.pdf]

Supplemental Data

Hydrogel Delivery Device for the In Vitro and In Vivo Sustained Release of Active rhGALNS Enzyme

Michael Flanagan [1], Qi Gan [1], Saahil Sheth [2], Rachel Schafer [3], Samuel Ruesing [2], Linda E. Winter [1], Karoly Toth [4], Silviya P. Zustiak [2,*] and Adriana M. Montaño [1,5,*]

1 Department of Pediatrics, School of Medicine, Saint Louis University, St. Louis, MO 63104, USA
2 Department of Biomedical Engineering, Saint Louis University, St. Louis, MO 63103, USA
3 School of Medicine, Saint Louis University, St. Louis, MO 63104, USA
4 Department of Microbiology and Molecular Immunology, School of Medicine, Saint Louis University, St. Louis, MO 63104, USA
5 Department of Biochemistry and Molecular Biology, School of Medicine, Saint Louis University, St. Louis, MO 63104, USA
* Correspondence: silviya.zustiak@slu.edu (S.P.Z.); adriana.montano@health.slu.edu (A.M.M.)

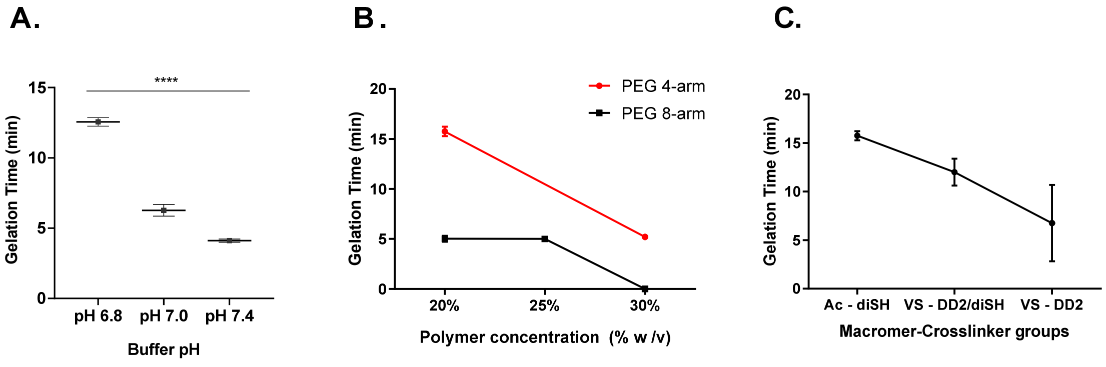

Figure S1: Effect of pH, polymer concentration and macromer:crosslinker on gelation time after mock subcutaneous injection (A) Hydrogel gelation time as a function of buffer pH for the hydrogel precursor solution. Hydrogels were prepared with 8-arm PEG-Ac macromer and PEG-diSH crosslinker and were 20% w/v in total polymer concentration. **(B)** Hydrogel gelation time as a function of polymer concentration. Hydrogels were prepared with PEG-diSH crosslinker at buffer pH 7.4. **(C)** Hydrogel degradation time as a function of macromer to crosslinker reactive groups. Hydrogels were prepared with 8-arm PEG-Ac macromer at pH 7.4 and were 20% w/v in total polymer concentration. Precursor solution was injected through a syringe needle prior to determining gelation time via inverted tube. Data are mean ± SD, n=4-8. **** $p \leq 0.0001$

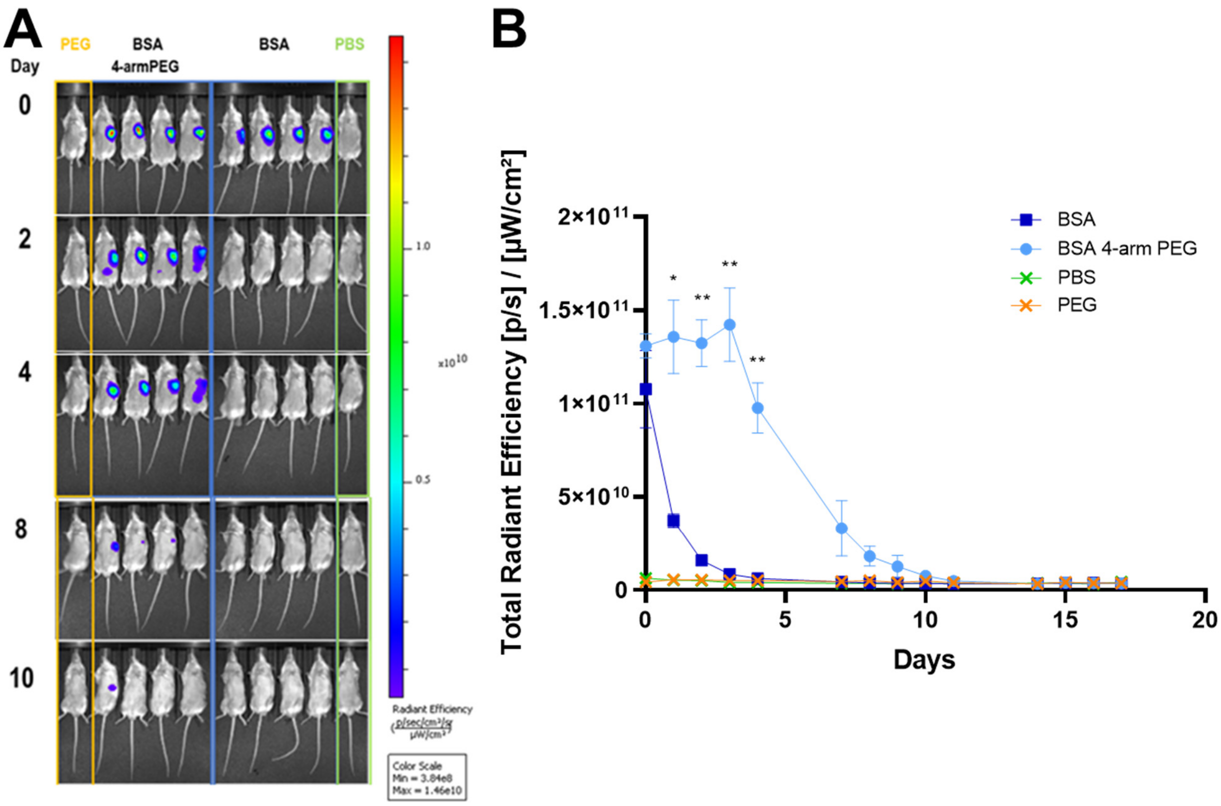

Figure S2: Representative whole body *in vivo* fluorescence images of live C57BL/6 albino mice with **(A,B)** non-encapsulated (n=4) or encapsulated (n=4) labeled-BSA in 4-arm PEG-Ac hydrogels. BSA at a dose of 10 mg/kg BW was administered subcutaneous (sub-Q) at day zero. Images were taken daily until 11 days post sub-Q injection.
